# Spontaneous Umbilical Hernia Rupture Associated With Omentum Evisceration in a Patient With Advanced Hepatic Cirrhosis and Refractory Ascites

**DOI:** 10.7759/cureus.16042

**Published:** 2021-06-29

**Authors:** Abdulqader M Albeladi, Ahmad M Odeh, Aminah H AlAli, Abdullah M Alkhars, Adeeb M Buhlaigah, Hussain A Alghadeer, Mohammed J Almosbeh, Mohmmed T AlAbbad, Mohammad S AlGhadeer

**Affiliations:** 1 Laparoscopic Surgery, Prince Saud Bin Jalawi Hospital, Al-Ahsa, SAU; 2 General Surgery, Prince Saud Bin Jalawi Hospital, Al-Ahsa, SAU; 3 Orthopaedics, King Fahad Hospital, Al-Ahsa, SAU; 4 Orthopaedics, King Faisal University, Al-Ahsa, SAU; 5 Pediatrics, Maternity and Children Hospital, Al-Ahsa, SAU

**Keywords:** flood syndrome, umbilical hernia, liver cirrhosis, refractory ascites, omentum evisceration

## Abstract

Flood syndrome is a spontaneous rupture of an umbilical hernia. It has a high mortality and morbidity and presents many challenges in medical versus surgical management. We present a case of a 23-year-old Yamani woman with complicated umbilical hernia, newly diagnosed hepatitis B infection, and decompensated liver cirrhosis with ascites (Child-Pugh grade B). The patient was undergoing multiple abdominal ascitic tapping that eventually ruptured with an omentum evisceration, causing Flood syndrome. An urgent umbilical hernioplasty with mesh in a sublay technique was conducted.

## Introduction

Ascites is a common complication of cirrhosis and indicates a new phase of hepatic decompensation in the progression of the cirrhotic process [1]. The mechanism that leads to the development of ascites in cirrhosis is multifactorial. Severe portal hypertension and hepatic insufficiency are the initial factors. A combination of increased intra-abdominal pressure from ascites and poor nutritional status then leads to the weakening of the anterior abdominal wall muscles, creating a defect [2]. Flood syndrome is described as a sudden gush of fluid from spontaneous umbilical hernia rupture. It is a life-threatening complication of chronic ascites and end-stage liver disease [3]. Estimated mortality rate is 30%. Up to 20% of patients with ascites in a hepatic cirrhosis setting develop umbilical hernias [2,3]. An umbilical hernia in these patients has a tendency to enlarge rapidly and to cause complications. Subsequently, it can rupture [4].

This case report presents the challenge in management of spontaneous rupture of an umbilical hernia which is a rare complication with high mortality rates and stresses the challenge of treatment that falls in the area between medical and surgical management.

## Case presentation

**Figure 1:**
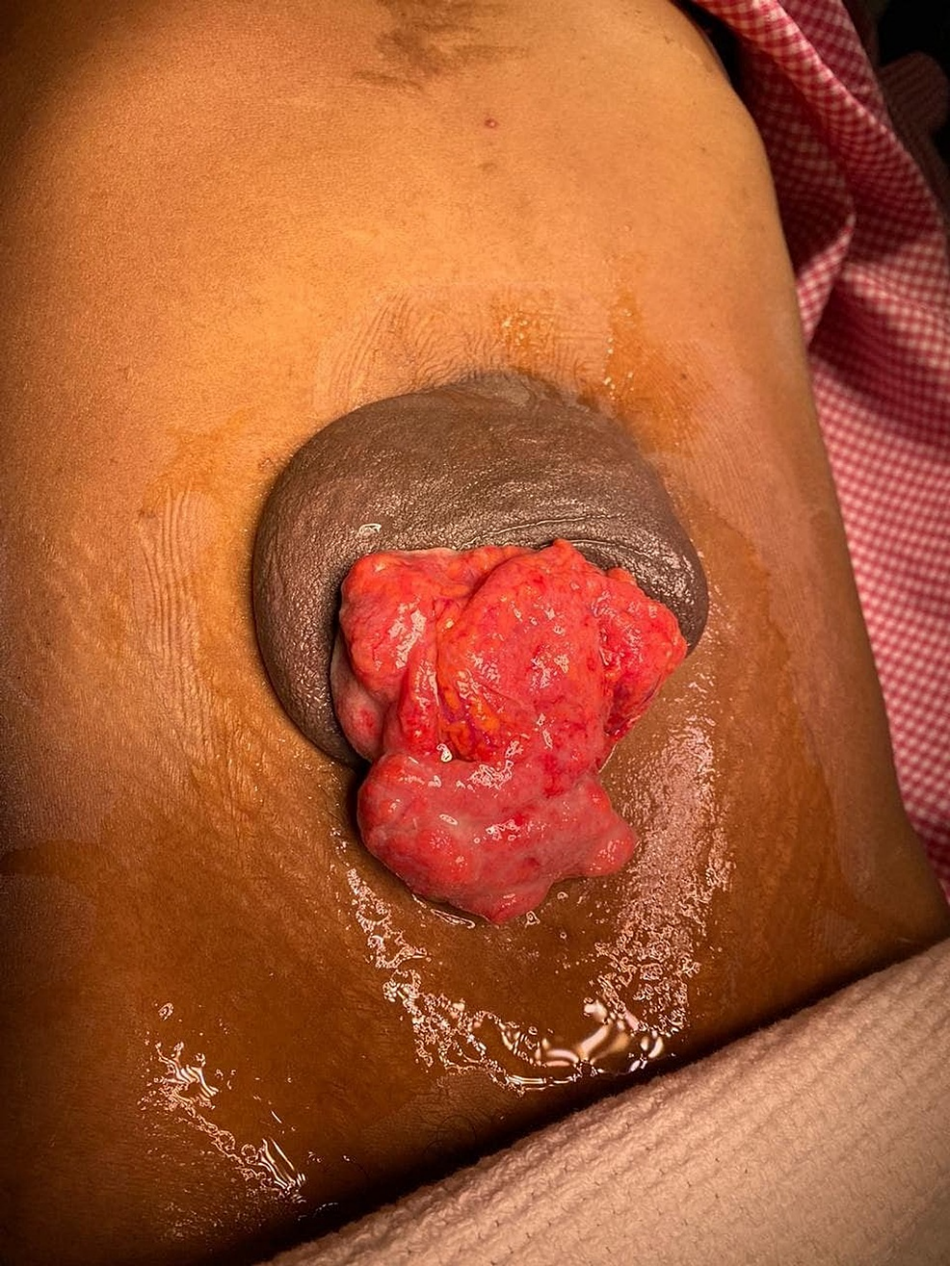
Spontaneous rupture of an umbilical hernia associated with omentum evisceration.

A 23-year-old Yamani woman presented to our emergency department with a case of abdominal pain and distension. Examination and investigations found an uncomplicated umbilical hernia, liver cirrhosis and ascites. The patient was admitted by the medical team, who investigated the patient for two weeks, eventually diagnosing a case of hepatitis B infection and decompensated liver cirrhosis (Child-Pugh grade B). After two weeks under medical care and multiple ascitic tapping, the patient responded with a sudden gush of clear fluid and spontaneous evisceration of the omentum from the umbilical hernia (Figure 1). The patient was referred from the medical team as a case of spontaneous umbilical hernia rupture and leaking ascitic fluid with omentum evisceration.

An urgent laparotomy was performed after preparation of the skin with an antiseptic solution. Excision of the umbilicus and eviscerated omentum was done. Aspiration of all ascitic fluid and samples were sent for cytology, biochemical, and microbiology testing. All abdominal viscera were examined, but no evidence of the recanalization of the umbilical vein was found.

After placement of an abdominal drain, a blunt dissection was done between the posterior rectus sheath and the rectus abdominus to facilitate placement of the polypropylene mesh in a sublay position. The posterior rectus sheath was closed using 1/0 non-absorbable polypropylene stitch. The mesh was placed over the posterior rectus sheath and below the rectus abdominus. The anterior rectus sheath was closed using 1/10 polypropylene stitch, and the skin was closed using clips.

Postoperatively, the patient stayed in the ICU for five days and was then moved to a regular ward. She received prophylactic broad spectrum antibiotics and albumin. Her abdominal drain output ranged from 1000-2000 ml clear ascitic fluid per day for one week, then decreased gradually over the next week to around 30 ml. The drain was removed, and the patient was discharged home. During follow-up in the outpatient clinic after two weeks, the patient was doing well with no wound complications. Consequently, the patient was referred to a tertiary center for further management.

## Discussion

The spontaneous rupture of an umbilical hernia, known as Flood syndrome, is a rare occurrence that can be accompanied by significant morbidity and mortality. It was first described in the literature in 1901 [2,3]. Complications of umbilical hernia rupture include an incarcerated bowel, spontaneous paracentesis and evisceration, hypotension, peritonitis, development of cellulitis, and sepsis [5]. Flood syndrome has high mortality and morbidity and presents many challenges in medical versus surgical management. The prevention of an umbilical hernia rupture depends on the optimal management of the underlying ascites in cirrhotic patients.

Conservative management includes the use of diuretics (furosemide and spironolactone), regular paracentesis, and non-steroidal inflammatory drugs with dietary salt and fluid restrictions [2]. Due to the difficulties Flood syndrome presents, surgical management is not a well-established procedure and is associated with a mortality rate of up to 30%, especially in patients undergoing emergent hernia repair. In cases of failed conservative management, the surgical options include umbilical herniorrhaphy, peritoneovenous shunting (PVS), transjugular intrahepatic portosystemic shunting (TIPS), and concomitant portal venous decompression [2,6]. However, postoperative mortality can be as high as 60% to 80% and morbidity up to 71%. Immediate surgical intervention decreases the mortality range to 6-20% [5]. Moreover, the postoperative control of ascites is still difficult concerning repair and the prevention of complications.

Elective herniorrhaphy is well recommended in cirrhotic patients with well-controlled ascites and no comorbidities [7]. In cases of a complicated hernia or uncontrolled ascites, however, the procedure is usually associated with postoperative complications [7]. However, urgent umbilical herniorrhaphy without mesh or primary closure is the preferred intervention in cirrhotic patients presenting an umbilical hernia rupture since it has been shown to reduce the mortality rate to 6-20% [2]. Our patient was treated with urgent surgical repair using mesh; some studies have reported instances of reduced hernia recurrence in patients who underwent mesh umbilical herniorrhaphy [8,9]. In our case, the patient was young and clinically stable with no signs of infection; hence, we decided to repair the hernia using the sublay technique because it has a lower rate of infection and recurrence [8,9]. The sublay technique can be used in elective repair and also in emergency situations [10,11]. Our patient, treated with an intraperitoneal drain, prophylactic broad spectrum antibiotics, albumin, and regular dressing, showed an improved abdominal distention and was discharged on day 14 in good condition. Finally, the postoperative control of ascites is mandatory to prevent recurrence and further complications.

## Conclusions

Flood syndrome is a spontaneous rupture of the umbilical hernia. It has a high mortality and morbidity and presents many challenges in medical versus surgical management. Elective hernioplasty in non-complicated cases is well recommended in cirrhotic patients with well-controlled ascites and no comorbidities. In complicated situations, urgent umbilical herniorrhaphy or primary closure is the preferred intervention. However, either in elective or emergency situations, mesh can be used with different techniques.
